# The association and mediation role of Food and Nutrition Literacy (FNLIT) with eating behaviors, academic achievement and overweight in 10–12 years old students: a structural equation modeling

**DOI:** 10.1186/s12937-022-00796-8

**Published:** 2022-07-01

**Authors:** Azam Doustmohammadian, Nasrin Omidvar, Nastaran Keshavarz-Mohammadi, Hassan Eini-Zinab, Maryam Amini, Morteza Abdollahi

**Affiliations:** 1 Gastrointestinal and Liver Diseases Research Center, Iran University of Medical Sciences, Tehran, Iran; 2 Department of Community Nutrition, Faculty of Nutrition and Food Technology, and National Nutrition and Food Technology Research Institute (WHO Collaborating Center), Shahid Beheshti University of Medical Sciences, Tehran, Iran; 3 School of Public Health and Safety, Shahid Beheshti University of Medical Sciences, Tehran, Iran; 4 Department of Nutrition Research, National Nutrition and Food Technology Research Institute (WHO Collaborating Center), Faculty of Nutrition and Food Technology, Shahid Beheshti University of Medical Sciences, Tehran, Iran

**Keywords:** Food and nutrition literacy, Eating behavior, Academic performance, Food security, Elementary school children, Structural Equation Model

## Abstract

**Background:**

Food and nutrition literacy is a key factor in shaping healthy dietary behaviors and may result in decreasing decrease the prevalence of overweight. Empirical research on food and nutrition literacy and its outcomes is limited, especially among children and adolescents. Thus, this study investigates the link between Food and Nutrition Literacy (FNLIT) with eating behaviors, academic performance, and overweight in 10–12 years old students in Tehran, Iran.

**Methods:**

This study was performed through two phases: 1) Proposing a conceptual model of the relationship between FNLIT and its determinants and outcomes, based on the existing evidence and previous models, and 2) Testing the proposed FNLIT model through a cross-sectional study on 803 primary school students (419 boys and 384 girls, from 34 public and 10 private primary schools), aged 10–12 years using structural equation modeling. Demographic, socio-economic, and household food security characteristics were collected by interviewing the students and their mothers/caregivers using a questionnaire. FNLIT was measured by a self-administered, locally designed, and validated questionnaire.

**Results:**

The fit indices suggested a reasonably adequate fit of the data to the hypothesized model (χ^2^/df = 2.03, *p* < 0.001, goodness of fit index (GFI) = 0.90, adjusted goodness of fit index (AGFI) = 0.88, comparative fit index (CFI) = 0.91, incremental fit index (IFI) = 0.91, root mean square error of approximation (RMSEA) = 0.04, standardized root mean residual (SRMR) = 0.06). SES was directly and positively related to FNLIT and its subscale in students. FNLIT score had a positive direct (non-mediated) relationship with healthy eating behavior and academic performance. This pattern was strongly reversed in unhealthy eating behavior. There was a full mediation relationship between FNLIT and overweight/obesity via healthy eating behaviors. SES predicted academic performance partially through the mediating effect of Food Label Literacy (FLL). The results indicated that despite the direct relationship between SES and academic performance, an indirect but negative relationship existed with food insecurity.

The finding also revealed the fully mediating role of Food Choice Literacy (FCL) in the relationship between demographic factors and healthy eating behaviors. Our study also found that Interactive Food and Nutrition Literacy (IFNL) protected unhealthy eating behaviors, and FCL predicted healthy eating behaviors in children.

**Conclusion:**

Our study draws attention to FNLIT, especially the skills domain, including IFNL, FCL, and FLL, as the most important determinant of healthy eating behavior, academic performance, and weight status in school-age children reduces social inequalities in children’s development.

To ensure an adequate level of FNLIT, educators should assess and plan to enhance food literacy skills in children and adolescents.

**Supplementary Information:**

The online version contains supplementary material available at 10.1186/s12937-022-00796-8.

## Introduction

Factors contributing to poor dietary habits are complex, and improving eating behavior requires an interdisciplinary approach that acknowledges the social context [1]. Among the different factors affecting eating behaviors, food literacy has recently been considered a key factor in improving diet quality, health, and well-being [2]. Food literacy is a construct that affects an individual’s ability to assess food and nutrition information, comprehend food labels, perform food safety precautions, use healthy cooking methods, apply dietary recommendations and make healthy food choices [3–5]. It may be a key factor in shaping dietary behaviors, specifically in children and adolescents [6, 7]. Healthy eating behaviors are essential in maintaining physical health and promoting optimal learning and school achievement [8]. Food literacy may contribute to a person’s ability to feed themselves (and others) in a nutrition-promoting way [9]. Enhancing food literacy to decrease nutrition-related disease burden is a growing international role for public health practitioners. However, assessing the impact of food literacy programming and the link between food literacy and health outcomes is hindered by the lack of validated measurement tools [10].

Doustmohammadian et al. developed a validated FNLIT questionnaire that measures the key functional, interactive, and critical elements of FNLIT in 10–12 years old students [11]. Guided by Nutbeam’s hierarchical model of health literacy [12], food and nutrition literacy in children is consisted of cognitive domains, including Understanding Food and Nutrition Information (UFNI) and Nutritional Health Knowledge (NHK) and skill domain including Functional Food and Nutrition Literacy (FFNL), Interactive Food and Nutrition Literacy (IFNL) and Critical Food and Nutrition Literacy (CFNL).

Childhood is a unique life stage through which decision-making skills are developed. Food and nutrition literacy programs are ideal for this stage because they focus on building capacity to operationalize healthy decisions [13]. There have been few studies on food and nutrition literacy improvement among adolescents [7]. Several conceptual models targeting the adult population have been suggested that improving food literacy might influence eating behavior and well-being [5, 14].

However, empirical research on food and nutrition literacy and its outcomes is limited [6, 15]. Thus, any relationship between food literacy/nutrition literacy and eating behaviors, food insecurity, academic achievement, or other health outcomes targeting children needs to be clarified to inform stakeholders involved in intervention design and curriculum content in this age group. Structural equation modeling (SEM) is a set of linear simultaneous equations considered an appropriate statistical technique for identifying the direct and indirect (through mediators) relationship between potential and observed variables [16, 17]. In SEM, individual differences and errors are also taken into account, which provides a more in-depth insight into the assessment of FNLIT, its determinants, and outcomes [18]. To date, no studies have used the SEM approach in this field. Therefore, the current study aimed to investigate the association between Food and Nutrition Literacy (FNLIT) components and eating behaviors, academic achievement, and weight status using the statistical approach of structural equation modeling (SEM), in elementary school children in Tehran, Iran.

### Conceptual framework

**Fig. 1:**
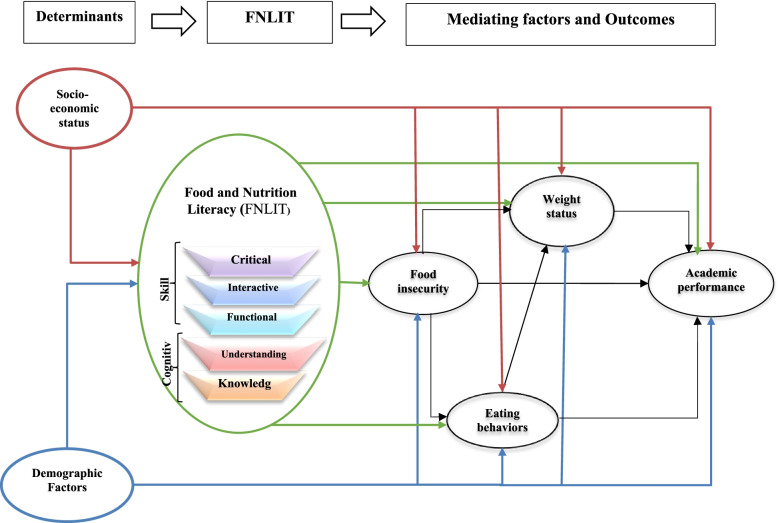
Proposed model of the relationship between FNLIT and its determinants and outcomes in 10–12 years old students. Arrows starting from the same determinant factors are in the same color

The conceptual framework of the study was proposed based on a review of prior evidence and previously described conceptual models on food/nutrition literacy (Fig. 1).

Linking food literacy/nutrition literacy to socio-demographic factors [19, 20], food security [21–23], weight status [24], and academic achievement [25] have been established in previous studies. The direct relationship between food or nutrition literacy and eating behaviors has been observed across several studies [6, 26, 27]. Chang et al. [28] theorized consumer capacities in the field of nutrition as factors that affect individuals’ food security and determined using a nutritional panel as a viable plan to reduce food insecurity. In addition, other studies focused on food literacy abstracted food security as a food literacy outcome [5, 6, 29–34]. They believed that the achievement of food literacy ensures access to a healthy diet. According to studies, food security is strongly related with children’s health and could affect anthropometrical indices [35, 36]. Other researchers have assessed the relationship between food insecurity, academic achievement, and anthropometric status in elementary school children [35]. On the other hand, Magulod et al. [37] reported anthropometric status and dietary behavior as determinants of academic achievement. The existing literature highlighted a moderate association between food consumption and behaviors on school performance among children [38].

Several proposed conceptual models or frameworks attempted to describe the relationship between food literacy, individual determinants, and outcomes related to health [5, 39–46]. Although food literacy has been presented as an essential factor for healthy food choices and a powerful resource to improve individual and public health, Vidgen and Gallegos (2014) found an indirect link between food literacy and healthy nutrition. They believe that food security and the ability to prepare food can enhance food choice and pleasure, stimulating healthy eating behavior [5]. In most conceptual frameworks, the authors have particularly focused on the sociocultural as well as environmental factors [5, 39–44].

## Methods

### Study design

This study used a population-based cross-sectional design, proposing a hypothesized model for explaining the relationship between FNLIT and its determinants and outcomes. Model fitness was addressed by testing the hypothesized conceptual model.

### Settings and sample

**Fig. 2:**
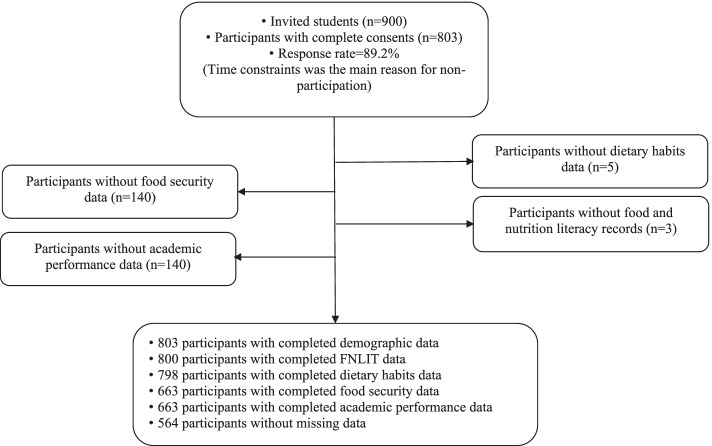
Study sampling frame

The study was performed from November 2015 to May 2017 in Tehran, the capital of Iran. The sampling frame can be seen in Fig. 2. The total eligible participants included 186,761 primary school children in the designated districts. Since there was no prior knowledge on the low FNLIT prevalence in primary school children to be estimated/examined for FNLIT, the *P*-value was 50% to obtain the highest P*(1-P) value for estimating the largest sample size. The sample size was determined to be at least 768 using the following formula, and 900 students were invited to increase statistical power further.

$$\mathrm{N}=\left(\mathrm{Z}1-\upalpha /2\right)2\ \mathrm{p}\ \left(1-\mathrm{p}\right)/\mathrm{d}2$$

Finally, 803 students aged 10 to 12 years completed the survey (response rate = 89.2%, power = 88%). Time constraints were the main reason for non-participation. According to the general office of education in Tehran, there are three socioeconomic levels in 19-educational-districts of Tehran, namely affluent (north), semi-affluent (center), and deprived (south). A list of primary schools within the boundary of the mentioned districts provided by education department was used to start sampling. The students were selected through a three-stage cluster random sampling technique. Initially, according to the population density of the districts, nine districts (three from each socio-economic level) were selected. In the second stage, private and public schools were randomly allocated in each district with a probability proportional to the target sample size of each school. Finally, the students were randomly chosen from the fifth and sixth grades (aged 10–12 years) of the selected schools, and the ultimate participants were chosen from 34 public and 10 private primary schools. The excluded subjects’ characteristics did not differ significantly from those in the study.

### Measurements

#### Food and nutrition literacy assessments

A valid self-administered questionnaire was applied to measure food and nutrition literacy in 10–12 years old students (details of its development and validation are explained elsewhere [11]). It was a 46-item questionnaire and included the cognitive and skill domains. Two sub-scales of Understanding Food and Nutrition Information (UFNI, 10 items) and Nutritional Health Knowledge (NHK, 5 items) were identified in the cognitive domain. The skill domain consisted of four sub-scales, including Functional Food and Nutrition Literacy (FFNL), Interactive Food and Nutrition Literacy (IFNL), Food Choice Literacy (FCL), and Critical Food and Nutrition Literacy (CFNL), encompassing ten, seven, six and four items each, respectively. Finally, Food Label Literacy (FLL) was evaluated by four dichotomous questions. Based on the characterized scores of low (≤51), medium (> 51- < 74), and high (≥74), the FNLIT score ranged between 25.8 and 96.8 [19].

#### Assessment of eating behavior

The research team developed the eating behavior questionnaire based on the previous questionnaires [8]. For qualitative content validity, a panel of six experts (three nutritionists, one health education and health promotion, one sociologist, and one social medicine) examined the initial questionnaire. Items were modified based on the experts’ comments. Then it was pretested on 30 students (15 boys and 15 girls) to assess the questions’ content and clarity, and the unclear items were reworded. Using the questionnaire, interviewers asked about the frequency of eating sweets and salty snacks, fast foods, energy-free beverages (water, coffee, tea), energy-containing beverages (soft drinks, juices), and meals during the previous week. The regularity of mealtime was evaluated according to frequency intake of breakfast, lunch, and dinner during the past 7-day and divided into four groups, including every day a week; 3–6 times a week; 1–2 times a week; and never. Consumption frequency of snacks was measured by a four-response scale: more than or equal to three times a day, twice a day, once a day, and never. The frequency of drinking water in a day was reported as four groups of ≥5 cups a day, 3–4 cups a day, 1–2 cups a day, and zero cup a day. The students were also questioned about the frequency of having drinks (e.g., tea/coffee/hot cacao, soft drinks, and fruit juices), fast foods (e.g., sausage/hamburger, pizza, fried potato, and restaurant foods/fast foods), and snacks (e.g., sweet and salty snacks) over the last 7 days. The possible responses included: ≥5 times a week, 3–4 times a week, 1–2 times a week, and never.

#### Food security status

The status of household food security was measured by the valid USDA’s Household Food Security Survey Module, which assessed the ability of the households to obtain and conserve food during the previous 12-month. The survey module was translated into Farsi and validated in the Iranian context [47, 48]. The USDA module consisted of 10 questions for households without and 18 questions for households with children and was completed face-to-face or by telephone with mothers. Based on the scale, the households were classified into four categories of food secure (FS) (raw score ranged between 0 and 2); food insecure (FI) without hunger (raw score ranged between 3 and 7); and food insecure (FI) with moderate hunger (raw score ranged between 8 and 12); food insecure with severe hunger (raw score ranged between 13 and 18). Due to the low prevalence of FI with moderate and severe hunger, they were pooled as one.

#### Academic achievement

The students’ teachers completed the author-designed questionnaire on academic achievement. Since children’s final grades were not available at the study time, this pretested, self-administered questionnaire was developed and used based on the teachers’ evaluations of the student’s achievement in class and with regard to the grades for mid-term assignments. The validity and consistency of the quantitative academic achievement were assessed using two evaluation approaches, including qualitative and quantitative. The teachers (*n* = 152) from 43 girls’ and boys’ primary schools assessed their students in math, reading, spelling, composition, science, and social-science using two scoring scales, including a quantitative scale of 0 to 20 points according to the grades attained in class and qualitative scale of 1 (very good) to 7 (very weak) points according to the teachers’ assessment of the student. The teachers were instructed to fill out the questionnaire.

#### Study covariates

Several baseline covariates, including age, birth order, family size, ethnicity, parental age, parental education, father’s job position, mother’s employment status, other income source(s) of family members, house ownership status, and receiving financial support were considered based on the available evidence [8, 49–51]. Anthropometric measurements were taken, and age-and sex- standardized BMIz-score was calculated based on World Health Organization Child Growth Standards (WHO AnthroPlus, 2007) [52]. The weight status of students was reported as thin (BMIz-score < −2SD), normal (BMIz-score ≥ −2SD and ≤ 1SD), overweight (BMIz-score > 1SD and ≤ 2SD) and obese (BMIz-score > 2SD). All participants (students and their parents) signed a written informed consent before the study.

The demographic and socioeconomic status were evaluated by a structured questionnaire through interviews with children and verified by their parents and/or caregivers after that. Parents completed the food security (FS) questionnaire through face-to-face interviews. Telephone interviews were conducted for those who could not attend schools (because of child care needs, illness, work constraints, etc.).

### Statistical analysis

the Kolmogorov–Smirnov test was used to assess the normal distribution of data. Categorical variables were presented as frequencies and percentages and compared using by ^χ2^ test. In the current study, the hypothesized model of direct and indirect associations among observed and latent variables was identified and evaluated through the following steps:

1. Exploratory Factor Analysis (EFA)

EFA was used to explore two latent constructs of healthy and unhealthy eating behaviors. Principal Component Analysis (PCA) with orthogonal Varimax rotation was applied to estimate factor loadings influencing observed variables. Items with an absolute loading of 0.30 remained in the structural model of healthy and unhealthy eating behaviors.

2. Confirmatory Factor Analysis (CFA)

The CFA method was applied to verify the measurement model of academic achievement and healthy and unhealthy eating behaviors by testing the association among the observed variables and their underlying latent construct (s).

3. Structural equation modeling (SEM)

SEM was used to examine the multidirectional relationships and causal dependencies between a number of endogenous and exogenous variables of interest [53]. SEM also allows the construction of latent variables, which are not measured directly and estimated from measured variables [53]. The latent variables were demographic, socio-economic status, healthy and unhealthy eating behaviors, and academic achievement in the current study. Measurable variables were FNLIT, BMIzscore, and food insecurity. We calculated standardized regression weights, standardized total effects, direct and indirect effects. Model fit measures were used to assess how well the proposed model captured the covariance between all the model items or measures. Goodness-of-fit to ensure that the proposed model can adequately explain the data was assessed by several model fit indices including χ2, the ratio of the χ2 to degrees of freedom (CMIN/DF), goodness of fit index (GFI), Adjusted Goodness of Fit Index (AGFI), the Comparative Fit Index (CFI), the incremental fit index (IFI), the Root Mean Square Error of Approximation (RMSEA) and Standardised Root Mean Residual (SRMR). CFI > 0.90, RMSEA and SRMR≤0.10, and CMIN/DF < 4.0 are considered to represent an appropriate model fit to the data. For GFI, AGFI, and NFI, which range from 0 to 1.0, values > 0.90 show an appropriate model fit to the data [54]. Statistical significance of the direct effects, indirect effects, and total effects were evaluated using a bootstrapping procedure. The number of bootstrapping was set at 2000 times.

Statistical analyses were performed using SPSS 21.0 (SPSS Inc., Chicago, Illinois, and the United States) and AMOS 21.0 [55]. All *P*-values were two-tailed, and P-values < 0.05 were considered statistically significant.

## Results

### Demographic and socio-economic characteristics of the study participants

A total of 803 students (419 boys and 384 girls) participated in the study. The mean age of students was 11.28 ± 0.65 years.

Girls and boys significantly differed in some demographic and socio-economic characteristics, including father’s education (*p* < 0.001), father’s job position (*p* < 0.001), and house ownership status (*P* = 0.005). Father illiteracy/low literacy (up to 5 years of education) was significantly more frequent in boys (15.5%) as compared to girls (5.5%). However, among boys, a higher proportion (21.6%) had fathers with higher-ranking job positions (employee/clerks) compared to girls (13.9%) (*p* < 0.001) and compared to girls (29%), a significantly higher proportion of the families of boys (36%) were tenants. Details on the background characteristics of the participants were described in Table 1.

**Table 1:** Baseline characteristics of 10–12 years old students in Tehran

Demographic characteristics	Total	Boys	Girls	***P*** value*
	N (%)	N (%)	N (%)	
**Grade**	803			0.59
fifth	413(51.6)	220(53.3)	193(46.7)	
sixth	390(48.4)	199(51.1)	191(48.9)	
**Birth order**	801			0.67
< 2	439(54.8)	232(55.5)	207(54)	
≥ 2	362(45.2)	186(44.5)	176(46)	
**Father age tertile (year):**	793			0.11
T1:30–40	210(26.5)	121(29.3)	89(23.4)	
T2:41–45	485(61.2)	236(57.1)	249(65.5)	
T3:≤ 46	98(12.3)	56(13.6)	42(11.1)	
**Mother age tertile (year):**	797			0.44
T1:23–35	25(3.1)	11(2.7)	14(3.7)	
T2:36–40	502(63)	263(63.5)	239(62.4)	
T3:≥ 41	270(33.9)	140(33.8)	130(33.9)	
**Ethnicity**	801			0.18
Fars	442(55.2)	217(51.8)	225(58.9)	
Azeri	230(28.7)	128(30.5)	102(26.7)	
Fars-Azeri	48(6)	25(6)	23(6)	
Other	81(10.1)	49(11.7)	32 (8.3)	
**School type**	803			0.59
Public	693(86.3)	359(85.7)	334(87.3)	
private	110(13.7)	60(14.3)	50(13)	
**Family size**	800			0.77
≥ 3	161(20.1)	82(19.6)	79(20.7)	
4	467(58.4)	249(59.6)	218(57.1)	
≤ 5	172(21.5)	87(20.8)	85(17.8)	
**Father education**	792			< 0.001*
illiterate to ≤5 years	85(10.7)	64(15.5)	21(5.5)	
6–9 years up to diploma	398(50.3)	210(50.8)	188(49.6)	
associate’s degree or higher	309(39)	139(33.7)	170(44.3)	
**Mother education**	797			0.22
illiterate up to ≤5 years	87(10.9)	52(12.6)	35(9.1)	
6–9 years up to diploma	463(58.1)	241(58.2)	222(58)	
associate’s degree or higher	247(31)	121(29.2)	126(32.9)	
**Father job position**	790			< 0.001*
Worker	106(13.4)	69(17)	37(9.9)	
Employee/clerk	327(41.4)	142(34.9)	185(49.6)	
high rank employee/clerk	140(17.7)	88(21.6)	52(13.9)	
Retired	20(2.5)	11(2.7)	9(2.4)	
self-manager	187(23.7)	97(23.8)	90(24.1)	
Unemployed	10(1.3)	4(1)	6(1.6)	
**Mother employment**	797			0.83
Working	633(79.4)	330(79.7)	303(79.1)	
Housewife	164(20.6)	84(20.3)	80(20.9)	
**Other income source of family members**	789			0.98
No	742(94)	388(92.6)	354(92.4)	
Yes	47(6)	24(5.7)	23(6)	
**House ownership status**	802			0.005^*^
Owner	428(53.4)	225(53.7)	203(53)	
Tenant	262(32.6)	151(36)	111(29)	
Mortgage	35(4.4)	16(3.8)	19(5)	
Other	77(9.6)	27(6.4)	50(13.1)	
**Financial support source**	802			0.25
No	784(97.7)	412(98.3)	372(97.1)	
Yes	18(2.2)	7(1.7)	11(2.9)	
**Z score for BMI**	803			< 0.001^*^
Thinness	15(1.9)	9(2.1)	6(1.6)	
Normal	382(47.6)	186(44.4)	196(51.0)	
Overweight	214(26.7)	97(23.2)	117(30.5)	
Obese	192(23.9)	127(30.3)	65(16.9)	
**HH**^**a**^ **food security status**	749			
FS^b^	563(75.2)	281(71.1)	282(73.4)	0.001^*^
FI^c^ without hunger	131(17.5)	88(22.3)	43(12.1)	
FI with hunger	55(7.3)	26(6.6)	29(8.2)	

^a^*HH* household

^b^*FS* food security

^c^*FI* food insecurity

*Significant at *p* < 0.05 for x^2^ tests

### FNLIT status of the study participants

FNLIT level of the participants is shown in Fig. S1. Approximately 11.6% of students were categorized as having low FNLIT, while almost 25% had low scores in FNLIT skill domain, and the majority scored moderate to high in cognitive domain (97.4%). Among subscales of FNLIT skill domain, a high proportion of students had low scores in critical FNLIT (42.2%) and food label literacy (81.1%). However, they scored better in food choice literacy (only 7.8% scored low).

### Confirmatory factor analysis

**Fig. 3:**
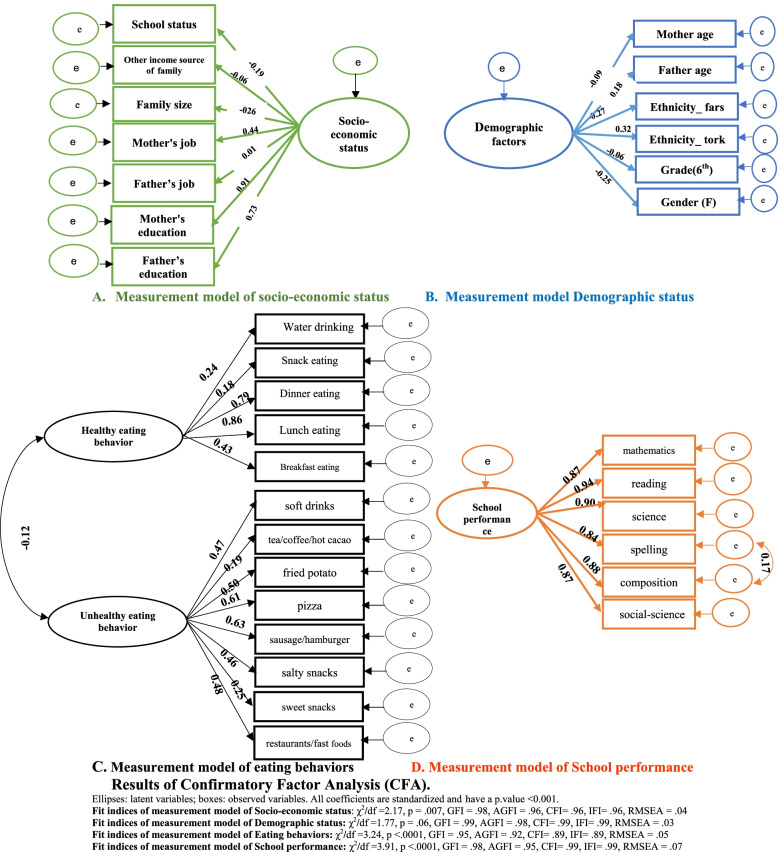
Results of Confirmatory Factor Analysis (CFA). Ellipses: latent variables; boxes: observed variables. All coefficients are standardized and have a p.value < 0.001. Fit indices of measurement model of Socio-economic status: χ^2^/df = 2.17, *p* = .007, GFI = .98, AGFI = .96, CFI = .96, IFI = .96, RMSEA = .04. Fit indices of measurement model of Demographic status: χ^2^/df = 1.77, *p* = .06, GFI = .99, AGFI = .98, CFI = .99, IFI = .99, RMSEA = .03. Fit indices of measurement model of Eating behaviors: χ^2^/df = 3.24, *p* < .0001, GFI = .95, AGFI = .92, CFI = .89, IFI = .89, RMSEA = .05. Fit indices of measurement model of School achievement: χ^2^/df = 3.91, *p* < .0001, GFI = .98, AGFI = .95, CFI = .99, IFI = .99, RMSEA = .07

The fit indices for CFAs used to develop the constructs of eating behaviors (χ2/df = 2.65, GFI = 0.95, AGFI = 0.93, CFI = 0.90, IFI = 0.90, RMSEA = 0.05) and academic achievement (χ2/df = 3.91, RMSEA = 0.07, GFI = 0.98, AGFI = 0.95, CFI = 0.99, IFI = 0.99, RMSEA = 0.07), socio economic factor (χ2/df = 2.17, *p* = 0.007, GFI = 0.98, AGFI = 0.96, CFI = 0.96, IFI = 0.96, RMSEA = 0.04) and demographic factor (χ^2^**/**df = 1.77, *p* = 0.06, GFI = 0.99, AGFI = 0.97, CFI = 0.99, IFI = 0.99, RMSEA = 0.03) had acceptable fit to the data. All components were significantly related to their latent variables (*p* < 0.001). Mother education and mother age explained a higher proportion of the variance of the socio economic and demographic factors, respectively. Reading mark, the frequency of eating lunch per week and the frequency of sausage/hamburger explained a higher proportion of the variance of academic achievement, unhealthy and healthy eating behaviors, respectively (Fig. 3).

### Structure equation modeling

**Fig. 4:**
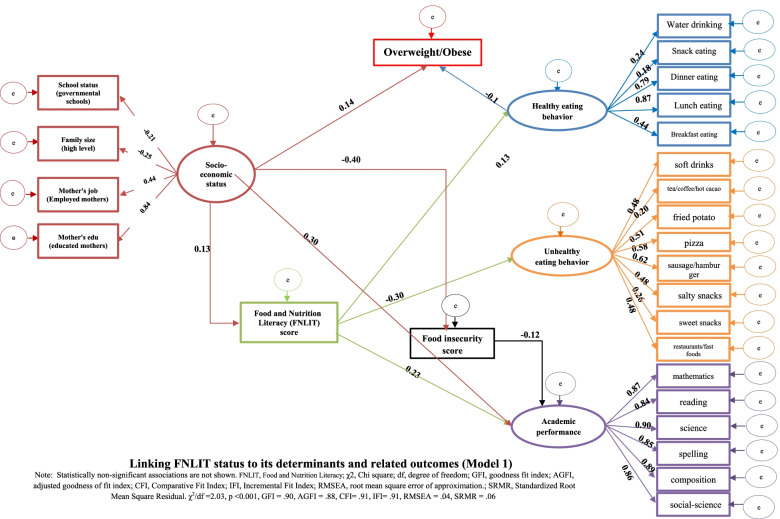
Linking FNLIT status to its determinants and related outcomes (Model 1). Note: Statistically non-significant associations are not shown. FNLIT, Food and Nutrition Literacy; χ2, Chi square; df, degree of freedom; GFI, goodness fit index; AGFI, adjusted goodness of fit index; CFI, Comparative Fit Index; IFI, Incremental Fit Index; RMSEA, root mean square error of approximation.; SRMR, Standardized Root Mean Square Residual. χ^2^/df = 2.03, *p* < 0.001, GFI = .90, AGFI = .88, CFI = .91, IFI = .91, RMSEA = .04, SRMR = .06

**Fig. 5:**
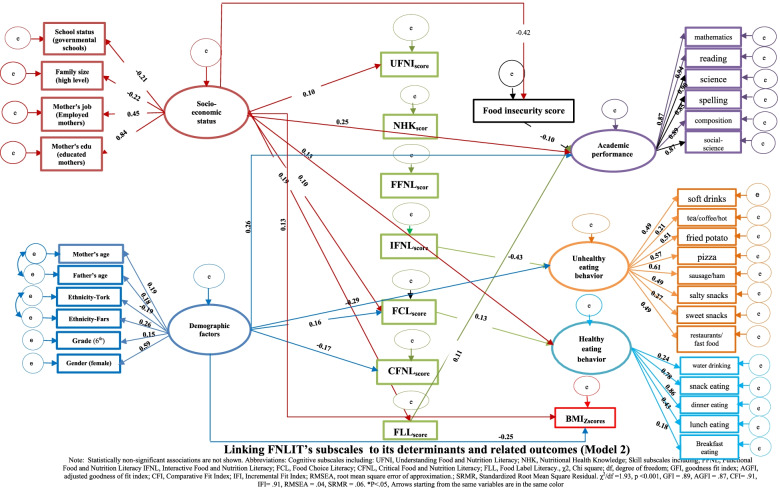
Linking FNLIT’s subscales to its determinants and related outcomes (Model 2). Note: Statistically non-significant associations are not shown. Abbreviations: Cognitive subscales including: UFNI, Understanding Food and Nutrition Literacy; NHK, Nutritional Health Knowledge; Skill subscales including; FFNL, Functional Food and Nutrition Literacy IFNL, Interactive Food and Nutrition Literacy; FCL, Food Choice Literacy; CFNL, Critical Food and Nutrition Literacy; FLL, Food Label Literacy., χ2, Chi square; df, degree of freedom; GFI, goodness fit index; AGFI, adjusted goodness of fit index; CFI, Comparative Fit Index; IFI, Incremental Fit Index; RMSEA, root mean square error of approximation.; SRMR, Standardized Root Mean Square Residual. χ^2^/df = 1.93, *p* < 0.001, GFI = .89, AGFI = .87, CFI = .91, IFI = .91, RMSEA = .04, SRMR = .06. *P < .05

The proposed FNLIT model was tested after controlling for the possible confounding impact of the background variables (Figs. 4 and 5). The fit indices suggested a reasonably adequate data fit for the hypothesized model. The standardized regression weights (β) from the structural equation model are shown in Models 1 and 2. When the direct and indirect effects of FNLIT on dietary habits were examined (Model 1, Table 2), FNLIT score had a positive direct (non-mediated) relationship with healthy eating behavior (β = 0.13, *P* = 0.005) and academic achievement (β = 0.23 *P* < 0.001). This pattern was strongly reversed in unhealthy eating behavior (β = − 0.30, *P* < 0.001). Socioeconomic status (SES), including mother’s job and education, school status (governmental), and the family size, was directly related to FNLIT score and overweight/obese in students (β = 0.13, *P* = 0.006; β = 0.14, *P* = 0.007 respectively), while also had direct (β = 0.30, *P* < 0.001) and indirect (food insecurity mediated) relationship with academic achievement (β = − 0.40, *P* < 0.001); β = − 0.12, *P* = 0.008 respectively). There was a full mediation relationship between FNLIT and overweight/obese via healthy eating behaviors. FNLIT indirectly through healthy eating behavior, had a negative effect on overweight/obese (of FNLIT on healthy eating behaviors, β = − 0.13, *p* < 0.01; of healthy eating behaviors on overweight/obese, β = −-0.10, *p* < 0.05) (Fig. 4, Table 2).

**Table 2:**
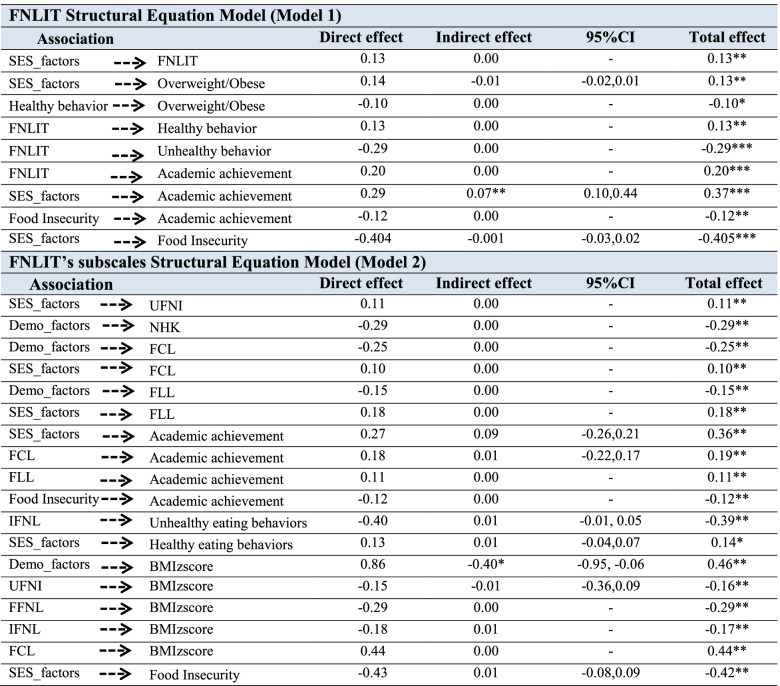
Standardized total effect, direct effect, and indirect effect in FNLIT Structural Equation Model (Model 1) and FNLIT’s subscales Structural Equation Model (Model 2) in 10–12 years old students in Tehran, Iran

*FNLIT* Food and Nutrition Literacy, *UFNI* Understanding Food and Nutrition Literacy, *NHK* Nutritional Health Knowledge, *FFNL* Functional Food and Nutrition Literacy, *IFNL* Interactive Food and Nutrition Literacy, *FCL* Food Choice Literacy, *CFNL* Critical Food and Nutrition Literacy, *FLL* Food Label Literacy, *CI* Confidence Intervals

*Significantly at *p* < 0.05, **Significant at *p* < 0.001, ***Significant at *p* < 0.01

Model 2 demonstrated the linking of FNLIT’s subscales to its determinants and related outcomes (Fig. 5). Mother’s job and education, school status (governmental schools), and family size were the socio-economic determinants of “Understanding Food and Nutrition Information” (β = 0.10, *P* < 0.05), “Food Choice Literacy” (β = 0.10, *P* < 0.01), and “Food Label Literacy” (β = 0.19, *P* < 0.01). SES predicted academic achievement partially through the mediating effect of “Food Label Literacy” (of SES on “Food Label Literacy”, β = 0.19, *P* < 0.01; of “Food Label Literacy” on academic achievement, β = 0.11, *P* < 0.05). The results indicated, despite the direct relationship between SES and academic achievement (β = 0.25, *P* < 0.001), an indirect but negative relationship existed through food insecurity (of SES on food insecurity, β = − 0.42, *P* < 0.001; of food insecurity on academic achievement, β = − 0.10, *P* < 0.05). Demographic factors negatively affected unhealthy eating behaviors (β = − 0.29, *P* = 0.001) and BMI_Z-score_ (β = − 0.25, *P* = 0.003).

Demographic factors, including mother’s age, ethnicity (fars), ethnicity (tork), grade, and gender, were the most important determinants of “Critical Food and Nutrition Literacy” (β = − 0.17, *P* = 0.01), FCL (β = 0.16, *P* = 0.02), unhealthy eating behaviors (β = − 0.29, *P* = 0.001), and BMI _Z-score_ (β = − 0.25, *P* = 0.003). The finding also revealed the fully mediating role of “Food Choice Literacy” in the relationship between demographic factors and healthy eating behaviors (of demographic factors on “Food Choice Literacy”, β = 0.16, *P* = 0.02; of “Food Choice Literacy” on healthy eating behaviors, β = 0.13, *P* = 0.02). In our study, we also found that “Interactive Food and Nutrition Literacy” protected unhealthy eating behaviors (β = − 0.43, *P* < 0.001), and “Food Choice Literacy” predicted healthy eating behaviors (β = 0.13, *P* = 0.02) in children.

The fit indices suggested a reasonably adequate fit of the FNLIT’s subscale model (Fig. 5).

## Discussion

In the current study, the proposed FNLIT conceptual model was tested to describe the determinants and outcomes of FNLIT in primary school children.

### Socio-economic determinants of the outcomes of FNLIT, eating behaviors, academic achievement, and overweight

Small family size, private schooling, mothers’ education, and occupation were the most important socio-economic determinants acting as the predictive factors of FNLIT and its subscales scores, including UFNI, FCL, and FLL. This finding is aligned with Sørensen et al., (2012) study, which reported the overall relationship between socio-economic status (parental education and occupation) and different forms of literacy (i.e., health literacy) in childhood and youth [56].

The evidence shows that family socio-demographic factors strongly influence children’s food literacy [57]. Parents, especially mothers, are important agents in the promotion of health, behavior, and abilities of their children; they create food environments and play a key role in structuring their children’s first experiences with food and eating through their own beliefs, food practices, perspectives, eating attitudes, knowledge, and understanding of the benefits of food and nutrients on health (1). The literature suggests that well-educated parents have a skill set that includes the ability to develop and facilitate children’s food and nutrition skills, for example, improving the critical thinking about media to defend from the persuasive influences of food advertisements (food and nutrition critical skills), opportunities to make food selections independently, such as by selecting a healthy snack at a convenience store after school using reading and understanding of food labels (food choice skills and food label literacy), obtain, interpret and apply of nutrition information (2, 3). The children of well-educated parents may better obtain, process, interpret and apply information that shapes their knowledge and attitudes about nutrition [58]. Parents can be role models for accessing and interpreting food and nutrition information and teaching children how to critically analyze the credibility and validity of information sources and media channels [59]. However, socioeconomic status has different impacts on food behavior and skills. Several studies have shown that communities with low socioeconomic status have more likely to make unhealthy food choices [60]. High socioeconomic status has also been linked with increased fruit and vegetables, dairy products, and other healthy foods among children and adolescents [58]. Families with a higher level of education may also promote greater investment in their children’s health. On the other hand, Larson et al. (2006) showed that greater involvement of children in food and nutrition skills/food literacy (food preparation and food shopping skill) is related to low socioeconomic status [24].

In addition to the significant direct effect of family SES on academic achievement, family SES indirectly affects academic achievement through FNLIT. The finding of several meta-analysis reviews [61, 62] revealed that parents’ location in the socioeconomic structure substantially impacts students’ academic achievement. Family SES sets the stage for students’ academic achievement by directly providing resources at home and indirectly providing the social capital necessary to succeed in school.

The current research has documented that those children with a low family size, educated and employed mothers, and children in the private schools had a higher probability of being overweight/obese. On the other hand, SES indirectly impacted overweight/obesity through FNLIT and healthy eating behaviors (negatively).

Based on the results, the effect of SES on academic achievement was mediated by food insecurity. Several studies have reported low SES and its connection to food insecurity as a key risk factor for unfavorable academic achievement [63–65]. Food insecurity has been associated with poor psychosocial outcomes, mental health, cognitive development, and compromised dietary intake, potentially resulting in malnutrition and, subsequently, low academic achievement [38, 64, 66].

Our results suggest that although SES is a condition that is difficult to change, helping children develop the cognitive and skill domain of FNLIT may improve their weight status and academic achievement and reduce social inequalities in child education and health.

The impact of socio-demographic characteristics on children’s skills (FCL and CFNL) can be explained that parents are more involved in daily food-related decisions and activities, including meal planning, grocery shopping, and cooking, that can create environments to influence the nutritional skills of children [67, 68]. Consistent with our results, models that focus specifically on children and young people emphasize the family’s demographic factors. The younger the child is, the more likely he/she is to rely on their parents' economic and social support [57, 69].

### Link between FNLIT, eating behaviors, academic achievement, and overweight

The relationship between healthy eating behaviors and overweight/obesity reduction has been shown in several studies [70, 71]. According to studies, by placing children at the center of food production, cooking food, selling food, and sharing food with their community, food literacy programs are helping to develop their food and nutrition skills to understand food and the benefits of a healthy diet. It will lead to a positive shift in healthy eating behaviors and may affect obesity trends [7, 72]. The current study also showed that FNLIT, especially in skills domain, is the essential determinant of healthy eating behavior. Regarding the association between FNLIT and eating behaviors, our findings are in line with interventional [73, 74] and correlational studies [75–77] as well as systematic review [6].

Previous studies found that high food literacy/nutrition literacy is associated with the frequency of main meal consumption [78], preference for healthy foods, lower intake of fast food and packaged or processed snacks among school-age children and adolescents in developed and developing countries [79, 80]. The limited awareness of FNLIT, functional, interactive, and food label literacy can create barriers to consuming a diverse and nutrient adequate diet [81]. Chung et al. (2017) stated that the unhealthy eating behaviors and lower quality of diet are related to the lack of food and nutrition knowledge, why and how food labeling information is read, and how healthy foods are prepared and safely saved to avoid food poisoning [26]. These are the so-called food and nutrition literacy concepts [5, 51].

Researches indicated that building food literacy in children through school gardens contributes to increased academic achievement, engagement, and self-confidence [82]. This relationship can be explained from two perspectives. First, FNLIT can reduce barriers to healthy eating and may influence adolescents’ dietary intake [6]. On the other hand, some studies confirm the association between eating behaviors and academic achievement in school-age children [83, 84]. Unhealthy eating patterns likely affect the intake of protein, vitamins, and minerals in both a quantitative and qualitative sense, jointly leading to metabolic consequences, which influence academic achievement negatively.

Adequacy of food choice literacy and food label literacy in children improves their ability to understand information about food items and food groups, read food labels, control their portions, and make informed decisions and healthy eating behaviors [9]. On the other hand, students with high critical skills are more involved in questioning and thinking critically to advance the various teaching fields [10]. These findings are consistent with our results on the mediating effect of food label literacy and food choice skills in promoting children’s eating behaviors.

The large study sample provided sufficient power to probe relatively small effects. In addition, this study enables us to minimize the confounding effects of other factors on the relations between FNLIT to eating behavior, academic achievement, and overweight/obesity by considering numerous covariates and their interactions using standardized regression analyses. To the best of our knowledge, the present study is the first to investigate the direct and indirect link between FNLIT, eating behaviors, academic achievement, and overweight in children. However, several limitations merit consideration. First, it is impossible to establish causal association due to the cross- sectional research design. However, SEM does let us evaluate the dose-response of the interrelationships of the variables. Second, our study population was not representative of the Iranian school population, meaning that our results may not be generalizable to the wider population of Iranian school children. The third is the memory bias that may occur when a self-reported dietary assessment method such as dietary recalls is applied. Furthermore, the impact of unmeasured confounders cannot be excluded entirely.

Future research with longitudinal design will be needed to elucidate the mechanisms involved in this relationship. Moreover, interventions to determine the relationship between change in food and nutrition literacy scores for before and after outcome variables are necessary.

## Conclusion

This research positions FNLIT within a conceptual model of its determinants and outcomes in primary school children and proposes an evaluation framework to guide investment and practice. These are critical foundations to further works in this field. The study results highlight groups within the school population who are at adequate levels of FNLIT, including having mothers with high-education levels, employed mothers, small family sizes, and children with private schooling children. These results serve as a general reminder that children (and their families) have highly varying educational needs that school educators and policymakers should consider new strategies based on this. In addition, our study draws attention to FNLIT, especially the skills domain, including IFNL, FCL, and FLL, as the essential determinant of healthy eating behavior, academic achievement, and weight status in school-age children and may reduce social inequalities in children’s development.

To ensure an adequate level of FNLIT, educators should assess and plan to enhance the food literacy skills of children and adolescents. Without a complete understanding of food, nutrition, and health, children and adolescents cannot gear up to improve their eating habits and diet quality. The current findings suggest that interventions targeting FNLIT improvement may effectively improve children’s eating behaviors, school academic achievement, and weight status. Stakeholders, including the government, food manufacturers, health providers, educators, and the food industry, should also play their roles to achieve a more significant reach and impact on the community.

## Supplementary Information

**Additional file 1: Fig. S1.** Food and nutrition literacy status in 10–12 years old students in Tehran.

## Data Availability

The datasets analyzed during the current study are available from the corresponding author on reasonable request.

## Abbreviations

FNLIT: Food and nutrition literacy
UFNI: Food and nutrition information
NHK: Nutritional health knowledge
FFNL: Functional food and nutrition literacy
IFNL: Interactive food and nutrition literacy
CFNL: Critical food and nutrition literacy
FCL: Food choice literacy
FLL: Food label literacy
SEM: Structural equation modeling
SES: Socioeconomic status
