# Supplementary material for: The association and mediation role of Food and Nutrition Literacy (FNLIT) with eating behaviors, academic achievement and overweight in 10–12 years old students: a structural equation modeling
Source: Nutr J. 2022 Jul 1;21:45. doi: 10.1186/s12937-022-00796-8 (PMC9248125; doi:10.1186/s12937-022-00796-8)
Supplement: Supplementary file 1 — Additional file 1: Fig. S1. Food and nutrition literacy status in 10–12 years old students in Tehran. [file 12937_2022_796_MOESM1_ESM.docx]

**Cognitive domain subscales**

**FNLIT and its domains**

**Skill domain subscales**

Fig S1: Food and nutrition literacy status in 10-12 years old students in Tehran
